# Performance of Different Gastric Cancer Screening Methods in Korea: A Population-Based Study

**DOI:** 10.1371/journal.pone.0050041

**Published:** 2012-11-29

**Authors:** Kui Son Choi, Jae Kwan Jun, Eun-Cheol Park, Sohee Park, Kyu Won Jung, Mi Ah Han, Il Ju Choi, Hoo-Yeon Lee

**Affiliations:** 1 National Cancer Control Institute, National Cancer Center, Goyang, Korea; 2 Department of Preventive Medicine & Institute of Health Services Research, College of Medicine, Yonsei University, Seoul, Korea; 3 Department of Epidemiology and Health Promotion, Graduate school of Public Health, Yonsei University, Seoul, Korea; 4 Department of Preventive Medicine, College of Medicine, Chosun University, Gwangju, Korea; 5 Gastric Cancer Branch, Research Institute and Hospital, National Cancer Center, Goyang, Korea; 6 Department of Social Medicine, College of Medicine, Dankook University, Chungnam, Korea; MOE Key Laboratory of Environment and Health, School of Public Health, Tongji Medical College, Huazhong University of Science and Technology, China

## Abstract

**Background:**

There is a lack of agreement on which gastric cancer screening method is the most effective in the general population. The present study compared the relative performance of upper-gastrointestinal series (UGIS) and endoscopy screening for gastric cancer.

**Methods:**

A population-based study was conducted using the National Cancer Screening Program (NCSP) database. We analyzed data on 2,690,731 men and women in Korea who underwent either UGIS or endoscopy screening for gastric cancer between January 1, 2002 and December 31, 2005. Final gastric cancer diagnosis was ascertained through linkage with the Korean Central Cancer Registry. We calculated positivity rate, gastric cancer detection rate, interval cancer rate, sensitivity, specificity, and positive predictive value of UGIS and endoscopy screening.

**Results:**

The positivity rates for UGIS and endoscopy screening were 39.7 and 42.1 per 1,000 screenings, respectively. Gastric cancer detection rates were 0.68 and 2.61 per 1,000 screenings, respectively. In total, 2,067 interval cancers occurred within 1 year of a negative UGIS screening result (rate, 1.17/1,000) and 1,083 after a negative endoscopy screening result (rate, 1.17/1,000). The sensitivity of UGIS and endoscopy screening to detect gastric cancer was 36.7 and 69.0%, respectively, and specificity was 96.1 and 96.0%. The sensitivity of endoscopy screening to detect localized gastric cancer was 65.7%, which was statistically significantly higher than that of UGIS screening.

**Conclusion:**

Overall, endoscopy performed better than UGIS in the NCSP for gastric cancer. Further evaluation of the impact of these screening methods should take into account the corresponding costs and reduction in mortality.

## Introduction

Gastric cancer is the fourth most common type of cancer (988,602 new cases, 7.8% of all new cancer cases in 2008) and the second most common cause of cancer death (737,419 deaths annually) in the world [1]. Some Asian countries, including China, Japan, and the Republic of Korea, have the highest gastric cancer incidences in the world [2]. Although the incidence of gastric cancer has declined in Korea in recent decades, it remains the most common cancer affecting the population [3].

Because the prognosis of early gastric cancer is favorable, high-prevalence countries have sought to reduce the disease burden by providing screening to average-risk populations. However, as gastric cancer screening is still fairly uncommon, and there is a paucity of data from Asia to support the establishment of gastric cancer screening programs [4], [5], [6], [7], [8], [9], [10]. Japan is one exception, and has been conducting mass gastric cancer screening using photofluorography (via indirect upper-gastrointestinal series) since 1960. Photofluorography screening is usually performed in a mobile van by the indirect x-ray method, in which a barium meal is used to make an image of the stomach. The early detection and consequent higher cure rates of gastric cancer and have led to a remarkable improvement in survival rates in Japan [6], [11], [12]. Cohort and case-control studies have also generally revealed a decreased risk of gastric cancer mortality in patients who have undergone photofluorography screening [5], [6], [13]. Although population-based photofluorography screening for gastric cancer has been mandated as a public policy matter in Japan, other opportunistic screening methods are also used in clinical settings throughout Asia, including endoscopy, serum pepsinogen testing, and *Helicobacter pylori* antibody testing [14].

Endoscopy is generally accepted as the gold standard for the diagnosis and clinicopathological evaluation of gastric cancer. Endoscopic examination has been predominantly used to screen symptomatic individuals, and to distinguish patients with gastric cancer from those with comparatively benign diseases, such as peptic ulcers. Moreover, less invasive endoscopy procedures, such as endoscopic mucosal resection and endoscopic submucosal dissection, have recently been established as treatments for early gastric cancer, without the risk of lymph node metastasis [15]. In recent years, endoscopy has been widely conducted as part of routine health check-ups. Some authors have reported higher detection rates of early gastric cancer with endoscopy than with UGIS. In a study conducted in Niigata, Japan, the detection of gastric cancer by endoscopy screening was about 2.7 times higher than that by direct or indirect radiography screening [16]. Despite promising results, the evidence regarding the effectiveness and complications of endoscopy screening, as well as the acceptance of individuals at average risk for gastric cancer to undergo the procedure, remains insufficient to justify its use in routine screening [17]. In Korea, a nationwide gastric cancer screening program was started in 1999 as part of the National Cancer Screening Program (NCSP) [18]. The NCSP recommends biennial gastric cancer screening for men and women aged 40 years or older, by either upper-gastrointestinal series (UGIS) or endoscopy [19].

Although the NCSP for gastric cancer offers participants the choice between UGIS and endoscopy screening, there is a lack of agreement on which method is most effective for screening in the general population. Thus, in the present study we estimated the performance of UGIS and endoscopy screening in the average-risk Korean population using the NCSP database. This study provides detailed estimates of key performance measures of UGIS and endoscopy screening: positivity rate, gastric cancer detection rate, interval cancer rate (ICR), sensitivity, specificity, positive predictive value (PPV), and stage distribution of screen-detected gastric cancer by screening method.

## Methods

### Study population

The NCSP for gastric cancer invites Medical Aid recipients and National Health Insurance (NHI) beneficiaries in the lower income brackets and aged 40 years or older to participate in gastric cancer screening. In 2002, NHI beneficiaries in the 20% income bracket were eligible for the program. In 2003, the NCSP expanded its target population to the 30% income bracket, and in 2005 the target population was further expanded to include NHI beneficiaries in the lower 50% income bracket. The population for the current study was restricted to individuals who participated in the NCSP for gastric cancer between January 1, 2002 and December 31, 2005. In total 2,318,558 participants underwent gastric cancer screening at least once during this time period. We excluded 54,804 participants (2.4%) due to missing screening results, 3,440 (0.1%) due to missing information on screening method, five because of incomplete identification numbers, and 9,917 (0.4%) due to a previous gastric cancer diagnosis according to the Korean National Cancer Incidence Database (KNCIDB), which contains 95% of newly diagnosed malignancies in Korea [20]. The final study sample consisted of 2,250,392 participants and 2,690,731 screening events.

The current study used the NCSP database, which includes participants' demographic characteristics and screening results, and written informed consent given by participants for the collection of their screening results and health data. We collected these data regularly from the NHI Corporation. For this reason obtaining informed consent for this specific study was waived due to the large size of the NCSP database. This study was approved by the institutional review board of the National Cancer Center, Korea.

### Gastric cancer screening and detection

Individuals invited to participate in the NCSP for gastric cancer could choose to undergo either UGIS or endoscopy screening at a clinic, or hospital designated as a gastric cancer screening unit by the NHI Corporation. Results of both screening methods were reported in seven categories (negative, peptic ulcer, benign tumor, possible gastric cancer, early gastric cancer, advanced gastric cancer, other). The results were defined as positive if the UGIS or endoscopy result was coded as possible gastric cancer, early gastric cancer, or advanced gastric cancer. Participants who had a positive screening result on UGIS were contacted by telephone by the medical staff of the gastric cancer screening unit, informed of the positive result and offered a follow-up examination. Follow-up examinations were performed within the framework of the NCSP. However, the NCSP database does not include diagnostic test results performed outside the program, as the NCSP database does not capture tests paid for privately, or conducted as a medical care service that is not part of screening.

Therefore, final gastric cancer diagnosis and tumor stage information were ascertained through linkage with the KNCIDB of the Korean Central Cancer Registry instead of using the NCSP database. Gastric cancer diagnoses reported to the KNCIDB through December 2006 were considered, to allow 12 months for any diagnostic work-up to be completed and the results to be fully reported. Tumor stages were recorded in the KNCIDB as localized, regional, distant, or unknown neoplasms, in accordance with the categories used in the Surveillance, Epidemiology, and End Results (SEER) Cancer Statistics Review of the United States National Cancer Institute [21].

### Statistical analyses

We calculated performance measures for each screening method. The positivity rate was calculated as the number of positive results per 1,000 screening events. Gastric cancer detection rate was calculated as the number of gastric cancer cases detected per 1,000 screenings. Interval cancers were defined as gastric cancer cases diagnosed outside the NCSP for gastric cancer within 1 year of a negative screening result in the NCSP. ICRs were calculated as the number of interval cancers per 1,000 screenings. Sensitivity was defined as the probability of a positive screening result, given a finding of cancer within 1 year of screening [true positive/(true positive+false negative)]. Specificity was defined as the probability of a negative screening result, given no finding of cancer within 1 year of screening [true negative/(true negative+false positive)]. The PPV was estimated as the number of screen-detected gastric cancer cases diagnosed per 100 positive screenings. We further analyzed the distribution of tumor stages by screening method and calculated the sensitivity of each screening method to detect localized, and regional or distant gastric cancer. All the performance measures were estimated by age group (40–49, 50–64, ≥65 years), gender, and health insurance status. Screening round was also considered in the calculations because positivity rates and cancer detection rates vary based on whether participants have previously undergone screening. The SAS software package (ver. 9.1; SAS Institute Inc., Cary, NC, USA) was used for all statistical calculations.

## Results

### Study population characteristics

A total of 2,690,731 screening events for gastric cancer took place between January 1, 2002 and December 31, 2005; approximately, 66% were UGIS screenings and 34% were endoscopy screenings. Endoscopy screening was chosen significantly more often by younger men, and NHI beneficiaries (*p*<0.001) (Table 1).

**Table 1:** Characteristics of 2,250,392 participants who underwent UGIS or endoscopy screening through the NCSP for gastric cancer, 2002–2005.

	UGIS^a^		Endoscopy^a^		Total	
	No.	%	No.	%	No.	%
Total No. of screening	1,765,909	65.6	924,822	34.4	2,690,731	100.0
Screening year						
2002	301,694	17.1	98,909	10.7	400,603	14.9
2003	396,713	22.5	172,760	18.7	569,473	21.2
2004	386,739	21.9	189,026	20.4	575,765	21.4
2005	680,764	38.6	464,127	50.2	1,144,891	42.5
Gender						
Men	675,128	38.1	379,324	41.0	1,054,452	38.2
Women	1,090,782	61.7	545,498	59.0	1,636,280	61.8
Age (years)						
40–49	503,454	28.5	341,348	36.9	844,802	31.4
50–59	522,239	29.6	3174049	34.3	839,643	31.2
60 and over	740,216	41.9	266,070	28.8	1,006,286	37.4
Health insurance type						
Medical Aid recipients	302,804	17.2	48,815	5.3	351,619	13.1
NHI beneficiaries	1,463,106	82.8	876,007	94.7	2,339,113	86.9

UGIS, upper-gastrointestinal series; NCSP, National Cancer Screening Program; NHI, national health insurance.

a All differences between participants who underwent UGIS screening and those who underwent endoscopy screening were statistically significant (*p*<0.001).

### Performance of gastric cancer screening methods

Of the 1,765,909 UGIS and 924,822 endoscopy screening, 70,049 and 38,969, respectively, were positive (Table 2). The positivity rate for endoscopy screening was statistically significantly higher than that for UGIS screening, except among men aged 40–49 and 50–59 years. The positivity rates of both UGIS and endoscopy screening were higher in the first screening round than in the subsequent round. Positivity rates also increased with increasing age, and were higher in men than in women.

**Table 2:** Positivity rate for gastric cancer by screening method, NCSP for gastric cancer, 2002–2005.

	UGIS			Endoscopy		
	No. of screening events	No. of positive results	Positivity rate per 1,000 (95% CI)	No. of screening events	No. of positive results	Positivity rate per 1,000 (95% CI)
Total	1,765,909	70,049	39.7 (39.7–40.0)	924,822	38,969	42.1 (41.7–42.6)
Screening round						
First round	1,484,579	60,403	40.7 (40.4–41.0)	765,813	32,589	42.6 (42.1–43.0)
Subsequent	281,330	9,646	34.3 (33.6–35.0)	159,009	6,380	40.1 (39.1–41.1)
Gender						
Men	675,128	37,699	55.8 (55.3–56.4)	379,324	18,524	48.8 (48.1–49.5)
Women	1,090,781	32,350	29.7 (29.3–30.0)	545,498	20,445	37.5 (37.0–38.0)
Gender, age (years)						
Men, 40–49	165,402	8,178	49.4 (48.4–50.5)	118,602	4,816	40.6 (39.5–41.8)
Men, 50–59	195,758	11,347	58.0 (56.9–59.0)	126,653	6,000	47.4 (46.2–48.6)
Men, 60+	313,968	18,174	57.9 (57.0–58.7)	134,069	7,708	57.5 (56.1–58.8)
Women, 40–49	338,052	8,864	26.2 (25.7–26.8)	222,746	7,627	34.2 (33.5–35.0)
Women, 50–59	326,481	9,763	29.9 (29.3–30.5)	190,751	7,337	38.5 (37.6–39.3)
Women, 60+	426,248	13,723	32.2 (31.7–32.7)	132,001	5,481	41.5 (40.4–42.6)
Health insurance type						
Medical Aid recipients	302,803	10,334	34.1 (33.5–34.8)	48,815	2,076	42.5 (40.7–44.4)
NHI beneficiaries	1,463,106	59,715	40.8 (40.5–41.1)	876,007	36,893	42.1(41.7–42.5)

UGIS, upper-gastrointestinal series; CI, confidence interval; NHI, national health insurance.

**Figure 1:**
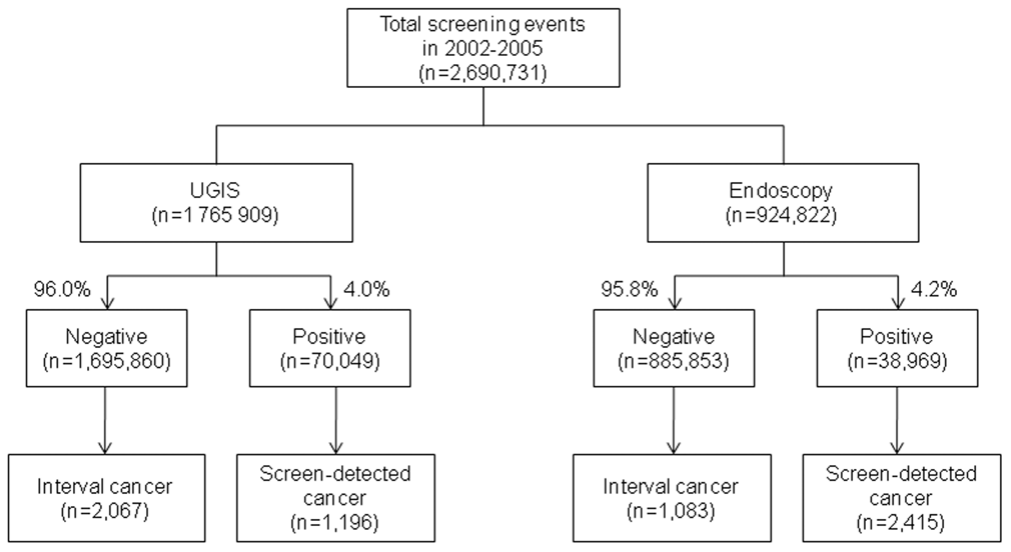
Diagram of gastric cancer screening in the NCSP.

Among participants with positive UGIS and endoscopy screening results, 1,196 and 2,415 gastric cancer cases were detected, respectively. The gastric cancer detection rate of UGIS screening was significantly lower (0.68 per 1,000 screenings) than that of endoscopy screening (2.61 per 1,000 screenings) (Table 3). However, the patterns between the two methods were similar; the cancer detection rate was higher in the first screening round than in the subsequent round, increased with age, and was higher in men and Medical Aid recipients. With regard to interval cancer, 2,067 and 1,083 interval cancers occurred within 1 year of a negative UGIS or endoscopy screening result, respectively (Figure 1). The ICRs also increased with age and were higher in men than in women. The ICR for UGIS screening (1.17/1,000 screenings) was not different from that of endoscopy screening (1.17/1,000 screenings).

**Table 3:** Detection rate and ICR for gastric cancer by screening method, NCSP for gastric cancer, 2002–2005.

	UGIS				Endoscopy			
	No. of cancer detected	Cancer detection rate per 1,000 (95% CI)	No. of interval cancers	ICR per 1,000 (95% CI)	No. of cancer detected	Cancer detection rate per 1,000 (95% CI)	No. of interval cancers	ICR per 1,000 (95% CI)
Total	1,196	0.68 (0.64–0.72)	2,067	1.17 (1.12–1.22)	2,415	2.61 (2.51–2.72)	1,083	1.17 (1.10–1.24)
Screening round								
First	1,068	0.72 (0.68–0.76)	1,726	1.16 (1.11–1.22)	2,074	2.71 (2.59–2.82)	914	1.19 (1.12–1.27)
Subsequent	128	0.45 (0.38–0.53)	341	1.21 (1.08–1.34)	341	2.14 (1.92–2.37)	169	1.06 (0.90–1.22)
Gender								
Men	830	1.23 (1.15–1.31)	1,370	2.03 (1.92–2.14)	1,660	4.38 (4.17–4.59)	767	2.02 (1.88–2.17)
Women	366	0.34 (0.30–0.37)	697	0.64 (0.59–0.69)	755	1.38 (1.29–1.48)	316	0.58 (0.52–0.64)
Gender, age (years)								
Men, 40–49	49	0.30 (0.21–0.38)	85	0.51 (0.40–0.62)	185	1.56 (1.34–1.78)	73	0.62 (0.47–0.76)
Men, 50–59	163	0.83 (0.71–0.96)	274	1.40 (1.24–1.57)	430	3.40 (3.07–3.72)	208	1.64 (1.42–1.87)
Men, 60+	618	1.97 (1.81–2.12)	1,011	3.22 (3.02–3.42)	1,045	7.79 (7.32–8.27)	486	3.62 (3.30–3.95)
Women, 40–49	40	0.12 (0.08–0.15)	81	0.24 (0.19–0.29)	160	0.72 (0.61–0.83)	59	0.26 (0.20–0.33)
Women, 50–59	58	0.18 (0.13–0.22)	146	0.45 (0.37–0.52)	224	1.17 (1.02–1.33)	93	0.49 (0.39–0.59)
Women, 60+	268	0.63 (0.55–0.70)	470	1.10 (1.00–1.20)	371	2.81 (2.52–3.10)	164	1.24 (1.05–1.43)
Health insurance type								
Medical Aid recipients	247	0.82 (0.71–0.92)	394	1.35 (1.21–1.48)	153	3.13 (2.64–3.63)	72	1.54 (1.18–1.90)
NHI beneficiaries	949	0.65 (0.61–0.69)	1,673	1.19 (1.13–1.25)	2,262	2.58 (2.48–2.69)	1,011	1.20 (1.13–1.28)

ICR, interval cancer rate; UGIS, upper-gastrointestinal series; CI, confidence interval; NHI, national health insurance.

### Sensitivity, specificity, and PPVs of gastric cancer screening methods

The respective sensitivities of UGIS and endoscopy screening were estimated at 36.7% and 69.0% (Table 4). The sensitivity of endoscopy screening was statistically significantly higher than that of UGIS. However, the specificity of UGIS and endoscopy screening were not significantly different: 96.1% and 96.0%, respectively. The sensitivity and specificity of UGIS and endoscopy screening were not associated with screening round, gender, age or health insurance type. The estimated PPV of UGIS screening (1.7%) was statistically significantly lower than that of endoscopy screening (6.2%). Overall, PPVs increased with age and were higher in men than in women.

**Table 4:** Sensitivity, specificity, and PPVs for detecting gastric cancer by screening method, NCSP for gastric cancer, 2002–2005.

	UGIS			Endoscopy		
	Sensitivity % (95% CI)	Specificity % (95% CI)	PPV % (95% CI)	Sensitivity % (95% CI)	Specificity % (95% CI)	PPV % (95% CI)
Total	36.7 (34.6–38.7)	96.1 (95.9–96.2)	1.7 (1.6–1.8)	69.0 (66.3–71.8)	96.0 (95.8–96.2)	6.2 (6.0–6.4)
Screening round						
First	38.2 (35.9–40.5)	96.0 (95.8–96.2)	1.8 (1.7–1.9)	69.4 (66.4–72.4)	96.0 (95.8–96.2)	6.4 (6.1–6.6)
Subsequent	27.3 (22.6–32.0)	96.6 (96.3–97.0)	1.3 (1.1–1.6)	66.9 (59.8–74.0)	96.2 (95.7–96.7)	5.3 (4.8–5.9)
Gender						
Men	37.7 (35.2–40.3)	94.5 (94.3–94.8)	2.2 (2.1–2.4)	68.4 (65.1–71.7)	95.5 (95.2–95.8)	9.0 (8.5–9.4)
Women	34.4 (30.9–38.0)	97.1 (96.9–97.3)	1.1 (1.0–1.3)	70.5 (65.5–75.5)	96.4 (96.1–96.6)	3.7 (3.4–4.0)
Gender, age (years)						
Men, 40–49	36.6 (26.3–46.8)	95.1 (94.6–95.6)	0.6 (0.4–0.8)	71.7 (61.4–82.0)	96.1 (95.5–96.7)	3.8 (3.3–4.4)
Men, 50–59	37.3 (31.6–43.0)	94.4 (93.9–94.8)	1.4 (1.2–1.7)	67.4 (61.0–73.8)	95.6 (95.0–96.1)	7.2 (6.5–7.8)
Men, 60+	37.9 (34.9–40.9)	94.4 (94.0–94.7)	3.4 (3.1–3.7)	68.3 (64.1–72.4)	95.0 (94.4–95.5)	13.6 (12.7–14.4)
Women, 40–49	33.1 (22.8–43.3)	97.4 (97.1–97.7)	1.1 (0.3–0.6)	73.1 (64.7–84.4)	96.6 (96.2–97.1)	2.1 (1.8–2.4)
Women, 40–59	28.4 (21.1–35.7)	97.0 (96.7–97.4)	0.6 (0.4–0.7)	70.7 (61.4–79.9)	96.3 (95.8–96.7)	3.1 (2.7–3.5)
Women, 60+	36.3 (32.0–40.7)	96.8 (96.5–97.1)	2.0 (1.7–2.2)	69.3 (62.3–76.4)	96.1 (95.6–96.6)	6.8 (6.1–7.5)
Health insurance type						
Medical Aid recipients	38.5 (33.7–43.3)	96.7 (96.3–97.0)	2.4 (2.1–2.7)	68.0 (57.2–78.8)	96.0 (95.2–96.9)	7.4 (6.2–8.5)
NHI beneficiaries	36.2 (33.9–38.5)	96.0 (95.8–96.1)	1.6 (1.5–1.7)	69.1 (66.3–72.0)	96.0 (95.8–96.2)	6.1 (5.9–6.4)

PPV, positive predictive value; UGIS, upper-gastrointestinal series; CI, confidence interval; NHI, national health insurance.

### Distribution of tumor stages by screening method

Staging of screen-detected gastric cancer is shown in Table 5. Localized cancer accounted for 32.4% and 45.7%, of gastric cancer cases detected by UGIS and endoscopy screening, respectively. To investigate which screening method was most sensitive for the detection of early gastric cancer, we categorized participants with gastric cancer into three groups according to tumor stage (localized, regional or distant, and unknown) and estimated corresponding sensitivities. The sensitivity of endoscopy screening to detect localized gastric cancer was 65.7%, which was statistically significantly higher than that of UGIS screening (32.1%).

**Table 5:** Sensitivity of difference screening methods to detect gastric cancer by stage, NCSP for gastric cancer, 2002–2005.

	UGIS		Endoscopy	
	Screen-detected gastric cancer No. (%)	Sensitivity % (95% CI)	Screen-detected gastric cancer No. (%)	Sensitivity % (95% CI)
Stage^a^				
Localized	388 (32.4)	32.1 (28.9–35.3)	1103 (45.7)	65.7 (61.8–69.5)
Regional or distant	381 (31.9)	37.8 (34.0–41.6)	543 (22.5)	73.6 (67.4–79.8)
Unknown	427 (35.7)	40.7 (36.9–44.6)	769 (31.8)	71.2 (66.2–76.2)

UGIS, upper-gastrointestinal series; CI, confidence interval.

a The following stage definitions were applied (adapted from the SEER Cancer Statistics Review); localized, a neoplasm confined entirely to the stomach without serosal involvement; regional, a neoplasm that extends beyond the limits of the stomach and invades the surrounding tissue; distant, a neoplasm that spreads to parts of the body remote from the primary tumor; unknown, a neoplasm with insufficient or unavailable information to assign a stage.

## Discussion

This study reported population-based data from a large gastric cancer screening program that allowed participants to choose either UGIS or endoscopy as a screening method. In the present study, only 34% of all gastric cancer screening was performed by endoscopy, with younger men being more likely to choose this method. However, the proportion of patients choosing endoscopy screening increased annually during the study period, from 24.7% in 2002 to 40.5% in 2005.

The positivity rate of endoscopy screening (42.1 per 1,000 screenings) was a little higher than that of UGIS screening (39.7 per 1,000 screenings). However, the gastric cancer detection rate of endoscopy screening (2.61 per 1,000 screenings) was 3.9 times higher than that of UGIS screening (0.68 per 1,000 screenings). The higher gastric cancer detection rate of endoscopy screening compared to UGIS screening was consistent across strata of screening round, gender, and age. These results are consistent with previous studies that compared the performance measures of endoscopy and UGIS screening [16]. A study conducted in Niigata, Japan, found that the gastric cancer detection rate of endoscopy screening was between 2.7- and 4.6-fold higher than that of UGIS or photofluorography screening [16], [17]. In agreement with previous reports, age and gender were associated with the likelihood of cancer detection in our study.

For the present analysis, we used data collected as part of the NCSP. Those who participated in the NCSP for gastric cancer during the period under study selected the screening method themselves, instead of being randomized. Therefore, differences in gender, age, and type of health insurance distribution appeared between those who underwent UGIS screening compared to endoscopy screening. The percentage of males and NHI beneficiaries was lower among those who underwent UGIS screening, but the percentage of participants aged 60 or over was higher when compared with endoscopy screening. Since the incidence of gastric cancer is higher in males, the elderly, and the lower income group [22], the demographic differences between those who underwent UGIS screening and endoscopy screening may have been counterbalanced. Actually, the estimated age-adjusted gastric cancer detection rate of endoscopy screening (2.69 per 1,000 screenings) was 3.5 times higher than that of UGIS screening (0.76 per 1,000 screenings) (data not shown). The higher gastric cancer detection rate of endoscopy screening may be partly explained by the fact that endoscopy can identify a lesion involving the mucosal surface of the stomach that conventional barium examinations may miss [23].

In the present study, UGIS screening failed to detect 2,067 of 3,263 gastric cancer cases (63.6%), and endoscopy screening failed to detect 1,083 of 3,498 cases (31.0%). These interval gastric cancer cases may have occurred as a result of an undetected abnormality at the time of screening (false-negative interval cancers) or as a new event after a negative screening result (true interval cancers) [24]. Unfortunately, this study could not distinguish false-negative from true interval cancers. However, as the doubling time of gastric cancer is approximately 2–3 years [25], most of the interval cancer cases in the present study were considered false-negative cases, and false-negative interval cancers were more likely to occur in participants who underwent UGIS screening than endoscopy screening.

We calculated ICR as the number of gastric cancer cases diagnosed within 1 year of a negative screening result per 1,000 screenings, and the ICR for UGIS screening was not different from that of endoscopy screening. However, the NCSP recommends biennial gastric cancer screening. Therefore we additionally estimated the ICRs of UGIS and endoscopy screening during the 2-year lapse between screening rounds. The ICRs for UGIS and endoscopy screening increased slightly as the lapse length increased; 1.89 (95% CI: 1.83–1.96) for UGIS screening, and 1.52 (95% CI: 1.44–1.60) for endoscopy screening (data not shown). The ICR for UGIS during the 2-year lapse between screening rounds was statistically significantly higher than that for endoscopy.

PPV is one of the most important screening program performance indicators, because high false-positive rates lead to a large number of unnecessary investigations, with a negative impact on cost-effectiveness. In the present study, the PPV of endoscopy screening was 6.2%, which was approximately 3.4 times higher than that of UGIS screening (1.7%). Several studies conducted in Japan have reported photofluorography PPVs between 0.78 and 2.03% [13], [26], [27], which is slightly higher than that of UGIS, but lower than that of endoscopy in this study.

Furthermore, the sensitivity of endoscopy screening (69.0%, 95% CI: 66.3–71.8) was higher than that of UGIS screening (36.7%, 95% CI: 34.6–38.7) in the current study. However, specificity was not statistically significantly different between the two screening methods. Previous studies have found that the sensitivity of photofluorography screening ranged from 56.8% to 88.5% [13], [27], [28], [29], whereas that of endoscopy screening fluctuated between 77.8% and 84.0% [30], [31]. Our sensitivity results were lower than those reported by others for both UGIS and endoscopy screening. However, it is difficult to directly compare the results of different studies due to differences in the target populations and in the gastric cancer screening programs themselves.

To compare the performance of UGI and endoscopy for detection of gastric cancer in population-based screening, we calculated the area under curve (AUC) using the summary receiver operating characteristic (SROC) curve. SROC curves were constructed using an estimated 1-specificity and sensitivity. We calculated AUC according to the Moses method, by extrapolating curves to the corners of SROC [32]. There is no absolute AUC threshold that defines a “good” test. However, an AUC of 1.0 defines a “perfect” test, and an AUC of 0.5 defines a “useless” test [33]. The AUCs of UGI and endoscopy were 0.6197 (95% CI: 0.4429–0.7965), and 0.9413 (95% CI: 0.9344–0.9482), respectively, and the AUC of endoscopy was statistically significantly higher than that of UGIS (p<0.001).

Tumor stage is an important prognostic factor following a diagnosis of gastric cancer. There is only very limited evidence to support the superiority of endoscopy screening over radiology screening methods for the detection of early gastric cancer. In this study, 45.7% of gastric cancer cases detected by endoscopy screening were localized, whereas the figure for UGIS screening was 32.4%. Furthermore, our data showed that endoscopy screening was statistically significantly more sensitive than UGIS screening to detect localized gastric cancer: 65.7% and 32.1%, respectively. Previous studies have reported that endoscopy screening was suitable for diagnosis of a lesion involving the mucosal surface of the stomach [23]. Nishizawa *et al* [34] stated that endoscopy was more effective than a barium meal study for the detection of early gastric cancer. Kubota *et al* [35] also found that endoscopy screening allowed for the detection of smaller gastric tumors than did radiography screening.

The present study has some limitations. First, we could not distinguish between symptomatic and asymptomatic participants. Those with symptoms would be more likely to have abnormal results and a diagnostic follow-up versus asymptomatic participants. Furthermore, there were substantial differences in the characteristics of participants who underwent UGIS screening and those who underwent endoscopy screening. These differences might have affected the positivity rate, gastric cancer detection rate, ICR and PPV, because these indicators depend on the prevalence of a given disease in the population. However, sensitivity and specificity, which were the key estimates of this study, are prevalence-independent test characteristics, as their values are intrinsic to the test and do not depend on the prevalence of a given disease in the population of interest. Therefore, the differences in characteristics between those who underwent UGIS screening and those who underwent endoscopy screening had a relatively small effect on sensitivity and specificity. Nevertheless, to minimize the confounding effect of non-randomization, all the performance measures were estimated by age group, gender, and health insurance type in the current study. We used health insurance type (Medical Aid vs. NHI) as a proxy for socioeconomic status. These indicators have been regarded as a highly reliable proxy for income. However, as we could not consider a participant's educational level or occupation as a proxy for socioeconomic status, it is possible that the socioeconomic status of participants was not fully reflected. Further, we couldn't consider histopathological features of the tumor such as histological differentiation, Lauren classification, and tumor location that might that might influence gastric cancer detection rate of UGIS and endoscopy screening. Second, although the NCSP is a population-based screening program, our study results may not be generalizable due to a low participation rate. Overall, approximately 13.4% of invited individuals were screened, and self-selection of participants cannot be excluded. However, it is not likely that these factors alone can explain the magnitude of the observed effect, and the overall detection rates reported in this study were comparable to those of other studies. Third, data available in the NCSP database does not include referral information or diagnostic test results conducted outside of the organized screening program (i.e. outpatient clinics, or private screening centers). Therefore, it was possibile that people who didn't attend follow-up within the NCSP were lost. To minimize loss to follow-up, we used the KNCIDB instead of the NCSP database to ascertain diagnosis of gastric cancer. The percentage of subjects in the KNCIDB that had to be identified by death certificate notification for 2003–200 was 4.7% for men and 4.3% for women, which shows the completeness of the KNCIDB [20], and suggests that it might be possible to disregard any bias due to loss to follow-up.

Overall, the better performance of endoscopy compared with UGIS screening supports the hypothesis that endoscopy screening may have a larger impact on gastric cancer mortality [16], [35], [36]. However, data on the impact of endoscopy screening programs on gastric cancer mortality are limited [35], [37], [38], [39]. Thus, further study is needed to determine whether endoscopy screening is more effective than radiography screening in reducing mortality. Although the data presented here are preliminary, our intermediate outcomes indicate that, in Korea, the introduction of endoscopy screening for gastric cancer in the average-risk population appears to perform better than UGIS screening. Further evaluation of the impact of these screening methods should take into account both cost and any associated reduction in gastric cancer mortality.

## References

[1] Ferlay J, Shin H, Bray F, Forman D, Mathers C, et al.. (2010) GLOBALCAN 2008, Cancer Incidence and Mortality Worldwide: IARC Cancer Base No. 10 [internet]. Lyon, france: International Agency for Research on Cancer.

[2] Jemal A, Bray F, Center MM, Ferlay J, Ward E, et al (2011) Global cancer statistics. CA Cancer J Clin 61: 69–90. 21296855 10.3322/caac.20107

[3] Shin H-R, Won Y-J, Jung K-W, Kong H-J, Yim S-H, et al (2005) Nationwide cancer incidence in Korea, 1999–2001: first result using the national cancer incidence database. Cancer Research and Treatment 37: 325–321. 19956367 10.4143/crt.2005.37.6.325 PMC2785938

[4] Lee KJ, Inoue M, Otani T, Iwasaki M, Sasazuki S, et al (2006) Gastric cancer screening and subsequent risk of gastric cancer: a large-scale population-based cohort study, with a 13-year follow-up in Japan. Int J Cancer 118: 2315–2321. 16331632 10.1002/ijc.21664

[5] Oshima A, Hirata N, Ubukata T, Umeda K, Fujimoto I (1986) Evaluation of a mass screening program for stomach cancer with a case-control study design. Int J Cancer 38: 829–833. 3793262 10.1002/ijc.2910380608

[6] Fukao A, Tsubono Y, Tsuji I, S HI, Sugahara N, et al (1995) The evaluation of screening for gastric cancer in Miyagi Prefecture, Japan: a population-based case-control study. Int J Cancer 60: 45–48. 7814150 10.1002/ijc.2910600106

[7] Hisamichi S, Sugawara N (1984) Mass screening for gastric cancer by X-ray examination. Jpn J Clin Oncol 14: 211–223. 6737710

[8] Inaba S, Hirayama H, Nagata C, Kurisu Y, Takatsuka N, et al (1999) Evaluation of a screening program on reduction of gastric cancer mortality in Japan: preliminary results from a cohort study. Prev Med 29: 102–106. 10446035 10.1006/pmed.1999.0507

[9] Mizoue T, Yoshimura T, Tokui N, Hoshiyama Y, Yatsuya H, et al (2003) Prospective study of screening for stomach cancer in Japan. Int J Cancer 106: 103–107. 12794764 10.1002/ijc.11183

[10] Pisani P, Oliver WE, Parkin DM, Alvarez N, Vivas J (1994) Case-control study of gastric cancer screening in Venezuela. Br J Cancer 69: 1102–1105. 8198977 10.1038/bjc.1994.216 PMC1969457

[11] Hisamichi S (1989) Screening for gastric cancer. World J Surg 13: 31–37. 2658351 10.1007/BF01671151

[12] Hisamichi S, Sugawara N, Fukao A (1988) Effectiveness of gastric mass screening in Japan. Cancer Detect Prev 11: 323–329. 3390854

[13] Abe S, Shibuya D, Noguchi T, Shimada T (2000) An estimate of the false-nagetive rate of mass-screening for gastric carcinoma. J Gastroenterol Mass Surv 38: 475–482.

[14] Hamashima C, Shibuya D, Yamazaki H, Inoue K, Fukao A, et al (2008) The Japanese guidelines for gastric cancer screening. Jpn J Clin Oncol 38: 259–267. 18344316 10.1093/jjco/hyn017

[15] Gotoda T (2007) Endoscopic resection of early gastric cancer. Gastric Cancer 10: 1–11. 17334711 10.1007/s10120-006-0408-1

[16] Tashiro A, Sano M, Kinameri K, Fujita K, Takeuchi Y (2006) Comparing mass screening techniques for gastric cancer in Japan. World J Gastroenterol 12: 4873–4874. 16937471 10.3748/wjg.v12.i30.4873 PMC4087623

[17] Leung WK, Wu MS, Kakugawa Y, Kim JJ, Yeoh KG, et al (2008) Screening for gastric cancer in Asia: current evidence and practice. Lancet Oncol 9: 279–287. 18308253 10.1016/S1470-2045(08)70072-X

[18] Choi KS, Jun JK, Lee HY, Park S, Jung KW, et al (2011) Performance of gastric cancer screening by endoscopy testing through the National Cancer Screening Program of Korea. Cancer Sci 102: 1559–1564. 21564421 10.1111/j.1349-7006.2011.01982.x

[19] Yoo KY (2008) Cancer control activities in the Republic of Korea. Jpn J Clin Oncol 38: 327–333. 18407932 10.1093/jjco/hyn026

[20] Won YJ, Sung J, Jung KW, Kong HJ, Park S, et al (2009) Nationwide cancer incidence in Korea, 2003–2005. Cancer Res Treat 41: 122–131. 19809561 10.4143/crt.2009.41.3.122 PMC2757659

[21] Young JL, Roffer SD, Ries LAG, Fritz AG, Hurlbut AA (2001) SEER Summary Staging Manual - 2000: Codes and Coding Instructions. Bethesda, MD: National Cancer Institute, NIH Pub.

[22] Kim CW, Lee SY, Moon OR (2008) Inequalities in cancer incidence and mortality across income groups and policy implications in South Korea. Public Health 122: 229–236. 17935744 10.1016/j.puhe.2007.07.003

[23] Hunt RH, Cotton PB, Crespi M, Drago JR, Kawai K, et al (1989) Role of endoscopy in the diagnosis of cancer: a consensus statement prepared by a working party of the International Union against Cancer. Cancer Res 49: 6822–6827. 2684402

[24] Woodman CB, Threlfall AG, Boggis CR, Prior P (1995) Is the three year breast screening interval too long? Occurrence of interval cancers in NHS breast screening programme's north western region. BMJ 310: 224–226. 7866124 10.1136/bmj.310.6974.224 PMC2548620

[25] Fujita S (1978) Biology of early gastric carcinoma. Pathol Res Pract 163: 297–309. 370808 10.1016/S0344-0338(78)80028-4

[26] Ishida T, Suematsu T, Oomayashi K, Takada Y, Kimura S, et al (1994) Measurement of accuracy of stomach mass screening by population-based cancer registration. J Gastrenterology Mass Surv 32: 9–16.

[27] Fukao A, Hisamichi S, Takano A, Sugawara N (1992) Accuracies of mass screening for gastric cancer-Teat sensitivity and program sensitivity. J Gastrenterology Mass Surv 97.

[28] Murakami R, Tsukuma H, Ubukata T, Nakanishi K, Fujimoto I, et al (1990) Estimation of validity of mass screening program for gastric cancer in Osaka, Japan. Cancer 65: 1255–1260. 2302674 10.1002/1097-0142(19900301)65:5<1255::aid-cncr2820650536>3.0.co;2-p

[29] Tsubono Y, Nishino Y, Tsuji I, Hisamichi S (2000) Screening for Gastric Cancer in Miyagi, Japan: Evaluation with a Population-Based Cancer Registry. Asian Pac J Cancer Prev 1: 57–60. 12718689

[30] Hosokawa O, Hattori M, Takeda T, Watanabe K, Fujita M (2004) Accurcy of endoscopy in detecting gastric cancer. Jpn J Gastroenterol Survey 42: 33–39.

[31] Otsuji M, Kouno Y, Otsuji A, Tokushige J, Shimotatara K, et al (1989) Assessment of small diameter panendoscopy for diagnosis of gastric cancer: comparative study with followup survey date. Stomach and Intestine 24: 1291–1297.

[32] Moses LE, Shapiro D, Littenberg B (1993) Combining independent studies of a diagnostic test into a summary ROC curve: data-analytic approaches and some additional considerations. Stat Med 12: 1293–1316. 8210827 10.1002/sim.4780121403

[33] Walter SD (2002) Properties of the summary receiver operating characteristic (SROC) curve for diagnostic test data. Stat Med 21: 1237–1256. 12111876 10.1002/sim.1099

[34] Nishizawa M, Nomoto K, Hosoi T, Okada T, Yamada K, et al (1982) Effectiveness of the small-diameter panendoscope in diagnosing cancers of the upper gastrointestinal tract. Endoscopy 14: 19–21. 7056239 10.1055/s-2007-1021565

[35] Kubota H, Kotoh T, Masunaga R, Dhar DK, Shibakita M, et al (2000) Impact of screening survey of gastric cancer on clinicopathological features and survival: retrospective study at a single institution. Surgery 128: 41–47. 10876184 10.1067/msy.2000.106812

[36] Dan YY, So JB, Yeoh KG (2006) Endoscopic screening for gastric cancer. Clin Gastroenterol Hepatol 4: 709–716. 16765306 10.1016/j.cgh.2006.03.025

[37] Ogura M, Hikiba Y, Maeda S, Matsumura M, Okano K, et al (2008) Mortality from gastric cancer in patients followed with upper gastrointestinal endoscopy. Scand J Gastroenterol 43: 574–580. 18415750 10.1080/00365520701813954

[38] Hosokawa O, Miyanaga T, Kaizaki Y, Hattori M, Dohden K, et al (2008) Decreased death from gastric cancer by endoscopic screening: association with a population-based cancer registry. Scand J Gastroenterol 43: 1112–1115. 18609154 10.1080/00365520802085395

[39] Riecken B, Pfeiffer R, Ma JL, Jin ML, Li JY, et al (2002) No impact of repeated endoscopic screens on gastric cancer mortality in a prospectively followed Chinese population at high risk. Prev Med 34: 22–28. 11749093 10.1006/pmed.2001.0925

